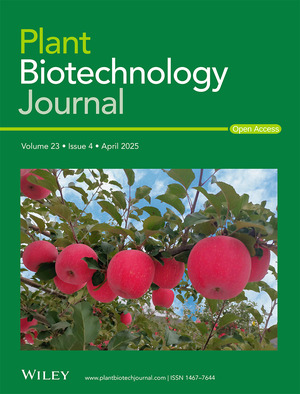# Issue Information

**DOI:** 10.1111/pbi.14390

**Published:** 2025-03-25

## Abstract

Front cover image:

A 61‐bp duplication in the promoter provided *MdNAC18.1* with an autosuppression module that regulates the overall tempo of fruit ripening through fine‐tuning ethylene biosynthesis. Cover illustration refers to the article published in this issue (Zhang et al., pp. 1216–1229).